# Membrane Transport Proteins in Osteoclasts: The Ins and Outs

**DOI:** 10.3389/fcell.2021.644986

**Published:** 2021-02-26

**Authors:** Amy B. P. Ribet, Pei Ying Ng, Nathan J. Pavlos

**Affiliations:** Bone Biology and Disease Laboratory, School of Biomedical Sciences, The University of Western Australia, Nedlands, WA, Australia

**Keywords:** osteoclast, membrane transporter, V-ATPase, bone disease, ion channel, CLCN7, solute carrier, osteoporosis

## Abstract

During bone resorption, the osteoclast must sustain an extraordinarily low pH environment, withstand immense ionic pressures, and coordinate nutrient and waste exchange across its membrane to sustain its unique structural and functional polarity. To achieve this, osteoclasts are equipped with an elaborate set of membrane transport proteins (pumps, transporters and channels) that serve as molecular ‘gatekeepers’ to regulate the bilateral exchange of ions, amino acids, metabolites and macromolecules across the ruffled border and basolateral domains. Whereas the importance of the vacuolar-ATPase proton pump and chloride voltage-gated channel 7 in osteoclasts has long been established, comparatively little is known about the contributions of other membrane transport proteins, including those categorized as secondary active transporters. In this Special Issue review, we provide a contemporary update on the ‘ins and outs’ of membrane transport proteins implicated in osteoclast differentiation, function and bone homeostasis and discuss their therapeutic potential for the treatment of metabolic bone diseases.

## Introduction

**FIGURE 1:**
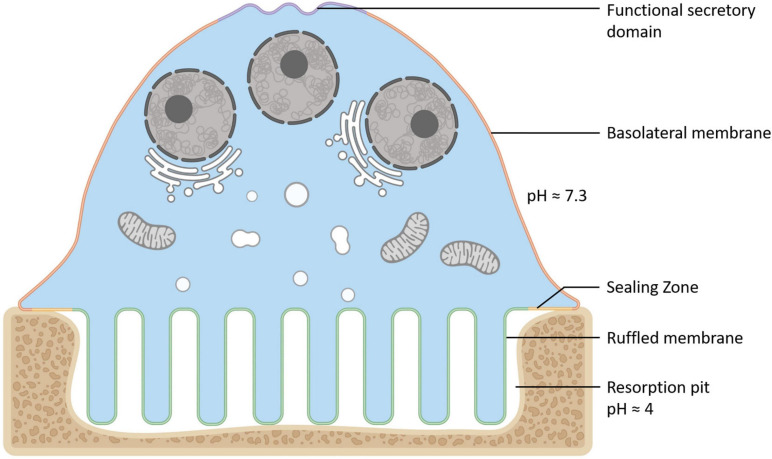
Anatomy of the Osteoclast. Illustration of the unique configuration and membrane organization of an osteoclast during active bone resorption. The osteoclast’s specialized plasma membrane domains are labeled and color-coded: Purple = the functional secretory domain, Orange = the basolateral membrane, Yellow = the sealing zone and Green = the ruffled border membrane. Created with BioRender.com.

Osteoclasts (OCs) are large bone-digesting (resorbing) cells that play a central role in the regulation of skeletal bone mass and bone pathologies such as osteoporosis. These multinucleated giants arise from the fusion of mononuclear progenitor cells of the monocyte/macrophage lineage in response to macrophage-colony stimulating factor (M-CSF) and receptor activator of nuclear factor-κB ligand (RANKL) (for review see Teitelbaum and Ross (2003)). Upon contact with mineralized bone, OCs adopt a polarized anatomy that denotes their active bone-resorbing status (Figure 1). First, the bone-facing ‘apical’ membrane is hermetically sealed to the bone surface via podosomes; a dense network of actin filaments connected to transmembrane adhesion proteins (e.g., β3-integrin). The membrane entrapped within this ‘sealing zone’, in turn, assumes a highly convoluted morphology termed the ruffled border. The ruffled border serves as the OC bone-resorbing apparatus and is formed upon the rapid fusion of secretory lysosomes with the bone-apposed plasmalemma (reviewed in Ng et al. (2019)). Thus, the composition of the ruffled border shares close analogy with endolysosomal membranes (Weivoda and Oursler, 2014; Roy and Roux, 2018) and together with the underlying resorptive space (hemivacuole or resorptive pit) is viewed akin to a giant digestive ‘extracellular lysosome’. Reflecting this, the resorptive hemivacuole is highly acidified (pH ∼4.5). Acidification is driven by the efflux of protons (H^+^) and chloride ions (Cl^–^) across the OC ruffled border membrane. This process is requisite to dissolve the inorganic phase of bone (hydroxyapatite) and the activation of acidic hydrolysases (chiefly cathepsin K) that digest the underlying organic collagen (Type 1a) matrix.

**FIGURE 2:**
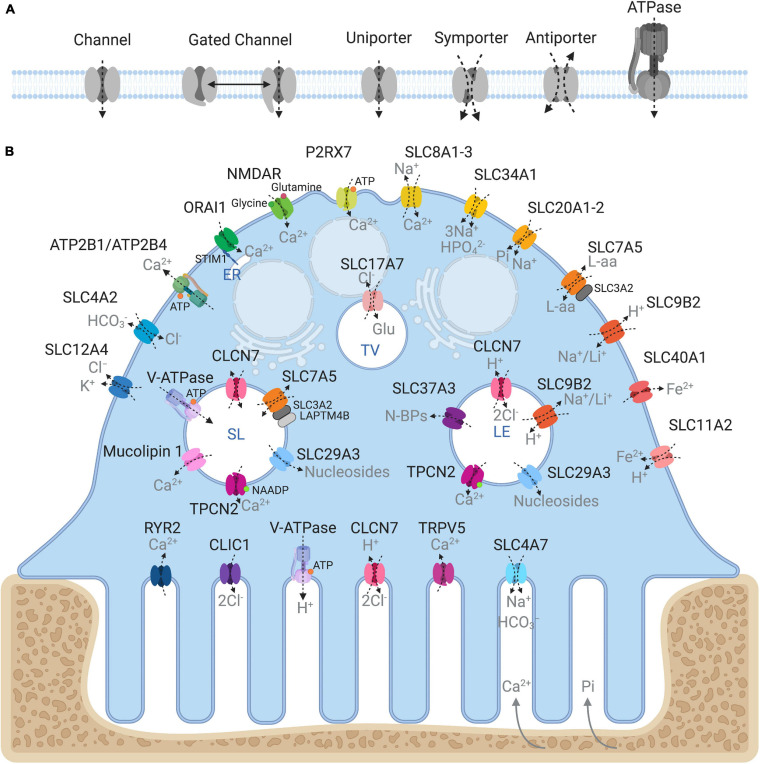
Ins and outs of membrane transport proteins in osteoclasts. **(A)** Schema depicting the major types of transport proteins and their modes of substrate movement across biological membranes. **(B)** Model summarizing the reported membrane localization of all the major membrane transport proteins expressed in osteoclasts. Known substrates are indicated in gray. Intracellular compartments correspond to: ER, endoplasmic reticulum; SL, Secretory lysosome; LE, Late endosome; TV, Transcytotic vesicle. Created with BioRender.com.

Being semipermeable, the ruffled border permits passive diffusion of small non-charged molecules (e.g., oxygen, carbon dioxide and water) across its membrane in-folds but restricts the influx of charged ions such as H^+^ and Cl^–^, leading to increased membrane potential and ionic pressure within the resorptive space. In addition, the dissolution of hydroxyapatite crystals (Ca_10_(PO_4_)_6_(OH)_2_) back into its elemental forms further exposes the ruffled border membrane to high ambient concentrations of calcium ions (Ca^2+^) and inorganic phosphate (Pi). In order to sustain OC cell volume and polarity, these resorption by-products must be removed and transported across the OC ruffled border and basolateral membranes. Some of these materials (e.g., degraded collagen) are internalized by bulk endocytosis at the ruffled border and transported apico-basolaterally via transcytotic carriers before being expelled at the functional secretory domain (FSD) (Nesbitt and Horton, 1997; Salo et al., 1997). By comparison, other by-products, such as ions and metabolites, are translocated by sets of structurally and functionally diverse membrane transport proteins, enriched on the opposing ruffled border and basolateral membranes (Figure 2). In their simplest form, membrane transport proteins are considered ‘gatekeepers’ of molecular exchanges across biological membranes and are operationally categorized according to their mode, direction and molecule of transport: i.e., (i) channels, (ii) gated channels, (iii) primary active transporters and (iv) secondary active transporters (uniporters, symporters and antiporters, inclusive) (Figure 2A). These integral transmembrane proteins can be further subdivided into four main superfamilies: (i) the ATP-binding cassette (ABC) transporters, (ii) ATPases, (iii) ion channels, and (iv) solute carrier proteins (SLCs) (reviewed extensively in (Pizzagalli et al., 2020)).

Collectively, membrane transport proteins not only facilitate complex and rich exchanges at the interface between the OC ruffled border or the basolateral surface with the extracellular milieu but also between membranes of intracellular organelles (such as lysosomes, the endoplasmic reticulum and mitochondria) and the cytosol (Figure 2B). In this way, they ensure that every functional unit of the OC receives its necessary complement of ions, metabolites and nutrients required for cellular homeostasis. In a giant energetic cell so heavily subjected to large ionic fluxes and membrane turnover, it is therefore unsurprising that an increasing number of mutations in membrane transport proteins have been implicated in the pathogenesis of bone diseases, in particular bone sclerosing (high bone mass) diseases associated with OC dysfunction (Table 1). For example, most of the known forms of OC-rich autosomal recessive osteopetrosis (ARO) have been linked to mutations in subunits of the multimolecular vacuolar-ATPase (V-ATPase) proton pump and chloride voltage-gated channel 7 (CLCN7), both indispensable components of the OC acidification machinery as described herein.

**TABLE 1:** Summary of known osteoclast membrane transport proteins, their localizations, substrates, associated disease phenotypes and impacts on osteoclasts.

					Knockout or knockdown	
					impacts OC function	
Name	Subcellular localization	Primary substrate	Human-Mouse protein sequence identity (%)	Known disease associations	*in vitro*	*in vivo*
V-type ATPase	Lysosome, Ruffled border	ATP, H^+^		Osteopetrosis (Frattini et al., 2000; Sobacchi et al., 2013)	Yes	Yes
CLIC1	Apical membrane	Cl^–^	98		No	No
CLCN7	Late endosome, Lysosome, Ruffled border	2Cl^–^, H^+^	96	Osteopetrosis, Hypopigmentation, organomegaly, and delayed myelination and development (Nicoli et al., 2019)	Yes	Yes
SLC12A4	Cell membrane	K^+^, Cl^–^	96		Yes	
SLC4A2	Basolateral membrane	Cl^–^, HCO_3_^–^	94			Yes
SLC4A7	Cell membrane	Na^+,^ HCO_3_^–^	74		Yes	
Ryanodine receptor 2	Cell membrane	Ca2^+^	97	Arrhythmogenic right ventricular dysplasia 2 (214) Catecholaminergic polymorphic ventricular tachycardia (215)		
ATP2B1	Basolateral membrane	Ca^2+^	99		Yes	
ATP2B4	Basolateral membrane	Ca^2+^	81	Variants may confer resistance to sever malaria (Timmann et al., 2012)	Yes	
TRPV4		Ca^2+^	95	Multiple neuromuscular disorders (Auer-Grumbach et al., 2010; Deng et al., 2010; Landouré et al., 2010)	Yes	
TRPV5	Ruffled border	Ca^2+^	81	Variant association with recurrent kidney stones (Palsson et al., 2019)		Yes
TRPV6		Ca^2+^	88	Transient neonatal hyperparathyroidism (Suzuki et al., 2018)		Yes
Mucolipin 1	Late endosome, Lysosome	Ca^2+^, Fe^2+^, Na^+^, K^+^, H^+^	91	Mucolipidosis IV (Boudewyn and Walkley, 2019)		Yes
ORAI1	Cell membrane	Ca2^+^	90	Immunodeficiency 9 (Feske et al., 2006). Tubular Aggregate Myopathy 2 (Nesin et al., 2014)	Yes	Yes
NMDA receptor	Cell membrane	Ca2^+^	99 (NMDAR1)	Neurodevelopmental disorder with or without hyperkinetic movements and seizures (Hamdan et al., 2011; Lemke et al., 2016)		
P2RX7	Cell membrane	Ca2^+^	80			Yes
TPCN2	Acidic organelles	Na^+^, Ca^2+^, H^+^	73	No link to disease but it has been linked to human pigmentation characteristics (Sulem et al., 2008)	Yes	
SLC8A1-3	Cell membrane	Na^+^, Ca^2+^	94, 94,96		Yes	
SLC34A1	Cell membrane	Na^+^, HPO_4_^2–^	90	Hypophosphatemic Nephrolithiasis/Osteoporosis 1 (Prié et al., 2002) Fanconi Renotubular Syndrome 2 (Magen et al., 2010) Infantile Hypercalcemia 2 (Schlingmann et al., 2016)		Yes
SLC20A1-A2	Basolateral membrane	Na^+^, Pi	93, 92	Idiopathic basal ganglia calcification (Wang et al., 2012; Jensen et al., 2013)		No
SLC37A3	Vesicles		89		Yes	
SLC7A5	Cell membrane, Lysosome	L-type amino acids	92		Yes	Yes
SLC9B2	Basolateral membrane	H^+^, Na^+^	81		No	No
SLC17A7	Synaptic vesicles	Glutamate	98			Yes
SLC29A3	Endosome, Lysosome	Nucleosides	74	H syndrome (Farooq et al., 2012) Dysosteosclerosis (Campeau et al., 2012)	Yes	
SLC40A1	Cell membrane	Iron	90	Hemochromatosis (Montosi et al., 2001; Njajou et al., 2001)		Yes (LysM-Cre)

*Human-Mouse protein sequence identity were obtained using BLAST.*

In this Special Issue, we briefly review the ‘ins and outs’ of membrane transport proteins in OCs. We highlight the contributions of established OC membrane transporters and channels (e.g., V-ATPases and CLCN7) as well as shed light on lesser-known membrane transport proteins whose physiological contributions in OCs and bone health have only just begun to emerge. We describe the structure, localization and function of these membrane transport proteins in OCs and bone as well as discuss their therapeutic utility as molecular targets for the treatment of metabolic bone diseases such as osteoporosis.

## Proton Transport in Osteoclasts: The V-ATPase Complex

The transport of protons (H^+^) across biological membranes is one of the most important physiological functions of cellular homeostasis, governing the regulation of intracellular pH, facilitating the generation of pH gradients in localized regions of cells, and maintaining the pH of the extracellular fluid. Aberrant regulation of pH in tissues and in cells leads to conditions such as acidosis and has been increasingly linked to the onset and progression of cancer (Parks et al., 2013; Aoi and Marunaka, 2014).

**FIGURE 3:**
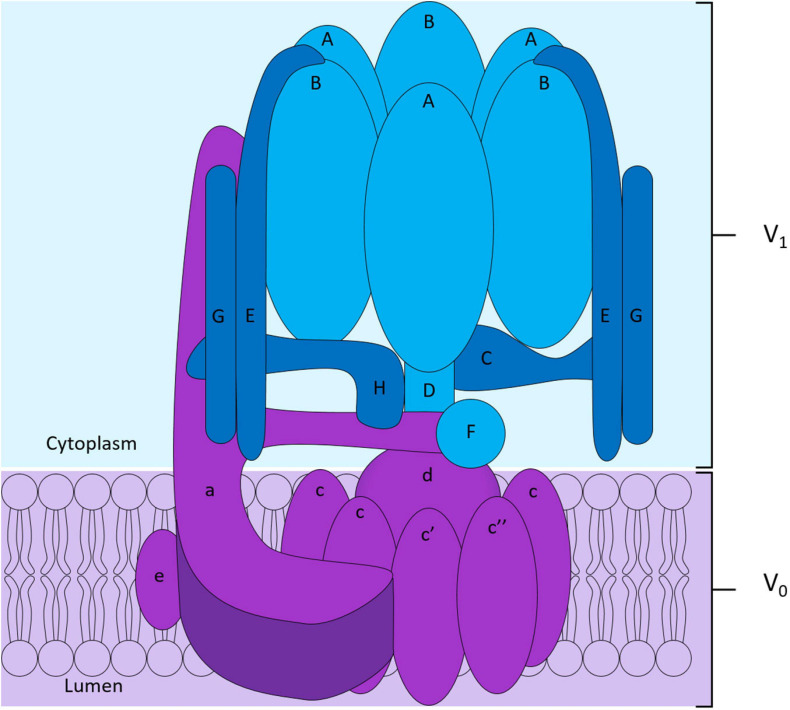
Structure of the V-ATPase proton pump. The V-ATPase complex is composed of two domains: the peri-membranous V_1_ domain composed of subunits A to H responsible for the hydrolysis of ATP shown in blue, and the intramembranous V_0_ domain who allows the translocation of protons across the membrane shown in purple in the diagram. The V0 domain is composed of the subunits a, e, d and of a hexameric ring formed by subunits c, c′ and c″.

To maintain intracellular pH homeostasis, cells have evolved sophisticated proton extrusion mechanisms and complementary buffering systems (e.g., CA2 and SLC4A2 reviewed below). Of these, the vacuolar H^+^-ATPase (V-ATPase) is among the most conspicuous features of eukaryotic endocytic and secretory organelle membranes and is arguably the keystone transporter for OC acidification and bone resorptive function. This large macromolecular complex is powered by the hydrolysis of ATP to extrude hydrogen ions (H^+^) across biological membranes. In OCs, the V-ATPase complex is enriched on the OC ruffled border membrane and decorates the surface of secretory lysosomes (Baron et al., 1985). Here, it facilitates the transport of protons necessary for acidification of the underlying resorptive space and activation of acidic hydrolysases (e.g., cathepsin K). Structurally, the V-ATPase proton pump is composed of 14 subunits and is the sum of two domains: (i) the ATP-hydrolytic domain (V_1_) and (ii) the proton-translocation domain (V_0_) (Figure 3). The V_1_ domain is composed of eight subunits (A to H) and is anchored indirectly to membranes through its interaction with the V_0_ domain. The V_0_ domain contains six subunits named a, d, e, c, c′, and c″, that assemble into a complex embedded into the membrane (Figure 3; Qin et al., 2012b; Duan et al., 2018). Following assembly, hydrolysis of ATP by the V_1_ domain generates the energy required to initiate V_0_ domain rotation. It is this active rotational movement that drives proton-translocation across the membrane (Forgac, 2007; Jefferies et al., 2008). For an extensive review see (Qin et al., 2012b).

The V-ATPase complex is obligatory for OC function and bone homeostasis. Accordingly, the gene expression levels of V-ATPase subunits are robustly amplified during RANKL-induced OC differentiation (Toyomura et al., 2000; Manolson et al., 2003). Transcriptionally, this is regulated by MITF (Microphthalmia-associated transcription factor) (Zhang et al., 2015), a transcription factor of the MiT-TFE family of basic helix-loop-helix leucine-zipper transcription factors that serve collectively as master regulators of lysosomal biogenesis, autophagy and osteoclastogenesis (Hershey and Fisher, 2004). Owing to its unique demands for acidification, the OC has also evolved cell-specific V-ATPase subunits. In particular, the OC-specific a3 subunit of the V_0_ domain encoded by *TCIRG1* in humans or *ATP6i* in mice, respectively. The a3 subunit has been shown to facilitate trafficking of the V-ATPase pump to the ruffled border (Sun-Wada et al., 2000; Futai et al., 2019) in collaboration with the small regulatory GTPases Rab7 and Rab27A (Matsumoto et al., 2018; Futai et al., 2019). Not surprisingly, mutations in *TCIRG1* account for ∼50% of OC-rich ARO in humans (Frattini et al., 2000; Sobacchi et al., 2013), a skeletal phenotype that is recapitulated in mice upon genetic ablation of *ATP6i* (Li et al., 1999).

## Neutralizing Membrane Potentials with Chloride Ions During Bone Resorption

The electrogenic efflux of H^+^ into the bone resorptive space generates a large membrane potential across the OC ruffled border (Kuno, 2018). To balance this charge, the OC must transport negatively charged counter-ions (anions) such as chloride (Cl^–^) across the ruffled border membrane. Failure to neutralize this membrane potential inhibits the acidification of the underlying resorption pit (Kornak et al., 2001). To achieve this, the OC furnishes its ruffled border membrane with complementary chloride-proton antiporters and secondary active transporters, described herein, that cooperatively facilitate Cl^–^ exchange across membranes.

## CLIC1

Chloride intracellular channel 1 (CLIC1) is a highly conserved chloride ion channel (Ulmasov et al., 2007) that exists intracellularly in a soluble state but undergoes reversible conformational changes that give rise to a dimeric integral membrane channel (Varela et al., 2019). The precise mechanisms governing CLIC1 membrane insertion remain unclear although oxidizing conditions, pH, Ca^2+^ and Zn^2+^ ion availability and membrane cholesterol have all been reported to influence its membrane integration (Valenzuela et al., 2013; Hossain et al., 2019). In OCs, the physiological role of CLIC1 has been contentious. Initial studies by Schaller et al. (2004) demonstrated that the chloride channel inhibitor NS3736 protected against ovariectomy-induced bone loss in rats owing to a dose-dependent inhibition of OC bone resorption. In keeping with this position, NS3736 was found to block OC acidification and bone resorption under *in vitro* settings. Based on these observations, the authors speculated that NS3736 inhibits the transport activity of either CLIC1 or CLCN7. Closer inspection of the expression levels of CLIC1 and CLCN7 revealed that whereas CLCN7 is highly expressed in OCs, CLIC1 is expressed widely in peripheral tissues, implying that NS3736 acted primarily on CLCN7 rather than CLIC1. Consistently, mice deficient in *CLIC1* lack an overt bone phenotype but rather exhibit bleeding abnormalities owing to platelet dysfunction (Qiu et al., 2010). Collectively, these data point to a physiological role for CLIC1 outside of the skeleton.

## CLCN7

Unlike CLIC1, the chloride voltage-gated channel 7 (CLCN7) (also known as CLC-7, OPTA2, and PPP1R63) is indispensable for OC function and bone homeostasis (Kornak et al., 2001). Intracellularly, CLCN7 exists as a homodimer residing on the membranes of late endosomes, lysosomes and the ruffled border where it functions as a 2Cl^–^/H^+^ antiporter with a fixed stoichiometry of 2Cl^–^ for each H^+^ transported (Graves et al., 2008). Unique among mammalian CLCN transporters, CLCN7 requires an obligatory β-subunit: osteoclastogenesis associated transmembrane protein 1 (Ostm1) (also known as CLCN7 accessory beta subunit) (Lange et al., 2006). This association is critical for the stability of CLCN7 and Ostm1 expression and Cl^–^/H^+^ exchange (Stauber and Jentsch, 2010). Similar to the V-ATPase proton pump subunits, the expression of both CLCN7 and Ostm1 is regulated by the transcription factor MITF (Meadows et al., 2007). CLCN7 traffics to late endosomes and lysosomes via interaction of its [DE]XXXL[LI] dileucine lysosomal targeting motifs with cognate adaptor proteins (Stauber and Jentsch, 2010). The mechanisms by which Ostm1 and CLCN7 cooperate on lysosome membranes has long remained unclear. However, recent cryoelectron microscopy studies by two independent groups demonstrated that the highly glycosylated Ostm1 serves as a molecular shield by covering the luminal surface of CLCN7 thereby protecting it from proteolysis in acidic environments that occur inside lysosomes and the resorptive space (Schrecker et al., 2020; Zhang et al., 2020). The importance of this intimate molecular association is exemplified in the skeleton where mutations in either CLCN7 or Ostm1 correspond with severe osteopetrosis in humans and in mice (Kornak et al., 2001; Chalhoub et al., 2003). The G215R mutation in CLCN7, for example, leads to autosomal dominant osteopetrosis type 2 that does not abolish the transport function of the channel but rather causes a severe trafficking defect with the G215R-CLCN7 mutation rendering CLCN7 retention in the ER (Schulz et al., 2010). OCs derived from both CLCN7 and Ostm1 mutant mice form normally but fail to form a ruffled border and resorb bone (Kornak et al., 2001; Chalhoub et al., 2003).

In addition to CLCN7, CLCN3 and CLCN5 have also been reported to reside on late endosomes in OCs (Okamoto et al., 2008) and mineralizing osteoblasts (Larrouture et al., 2015). Genetic ablation and siRNA-mediated depletion of *Clcn3* was found to reduce the acidification and bone resorptive capabilities of mouse OCs cultured *in vitro* (Okamoto et al., 2008). *Clcn3* knockout mice lack a bone phenotype although, like their *Clcn7* knockout counterparts, they exhibit severe neurodegeneration (Yoshikawa et al., 2002; Kasper et al., 2005; Okamoto et al., 2008). This indicates that CLCN3 is dispensable for OC function and skeletal homeostasis.

## SLC12A4

Outside the CLCN family, solute carrier family 12 member 4 (SLC12A4), also known as the electroneutral potassium-chloride cotransporter 1 (KCC1), has been shown to facilitate Cl^–^ extrusion in OCs. SLC12A4 localizes exclusively to the OC basolateral surface where it has been proposed to function in the cotransport of K^+^ and Cl^–^ simultaneously in the outward direction. Unlike other members of the SLC12 family, SLC12A4 K^+^/Cl^–^ cotransport is not dependent on the presence of Na^+^ (Hebert et al., 2004). Structurally, SLC12A4 is composed of 1085 amino acids forming a 12 transmembrane domain structure with a large extracellular loop containing potential N-linked glycosylation sites, and cytoplasmic N- and C-terminal domains (Gillen et al., 1996). To enable transporter function, SLC12A4 dimerises through links in both transmembrane and extracellular domains (Liu et al., 2019). Although ubiquitously expressed, the function of *Slc12a4* has been explored in mouse OCs in *in vitro* settings (Kajiya et al., 2006). Studies by Kajiya et al. (2006) demonstrated that Slc12a4 is the primary potassium-chloride transporter expressed in OCs. Moreover, treatment of OCs with the KCC transporter inhibitor R(+)-butylindazone (DIOA), or knockdown of *Slc12a4* expression using siRNA, decreased Cl^–^ extrusion resulting in increased OC alkalinisation and, in turn, reduced capacity to resorb bone (Kajiya et al., 2006). It remains to be seen whether these *in vitro* defects in OC activity extend to physiological impacts on the skeleton in intact mice.

## Internal Buffering and HCO_3_^–^ Transport in Osteoclasts

To sustain acidification during ongoing cycles of bone resorption, the V-ATPase pump requires a continuous supply of protons. Protons are supplied to the V-ATPase pump by the cytoplasmic carbonic anhydrase 2 (CA2), an enzyme that catalyses the reversible hydration of carbon dioxide along the following equation: CO_2_ + H_2_O → H^+^ + HCO_3_^–^. Unsurprisingly, defects in CA2 activity lead to osteopetrosis and extra-skeletal disturbances including renal tubular acidosis and cerebral calcification, reminiscent of the clinical features observed in patients harboring mutations in *TCIRG1*. At the same time, CA2 stabilizes intracellular pH (pHi) and alkalinisation in OCs through the liberation of HCO_3_^–^ ions. HCO_3_^–^ ions are, in turn, exchanged for Cl^–^ by select members of the solute carrier protein family 4 (SLC4), thereby ensuring an electroneutral supply of Cl^–^ to the OC (Teti et al., 1989).

## SLC4A2

SLC4A2, previously known as EPB3L1 or AE2, is a member of the SLC4 family of Cl^–^/HCO_3_^–^ exchangers. SLC4 family members are important for the modulation of pHi and have been implicated in the regulation of cell volume, migration, and transepithelial movement of ions in various tissues (Alper, 2006). The SLC4 family comprises 10 members divided into three major clades; (i) the Na^+^-independent electroneutral Cl^–^/HCO_3_^–^ exchangers (e.g., SLC4A1), (ii) the Na^+^-dependent HCO_3_^–^ transporters (e.g., SLC4A4) and, (iii) borate transporters comprised of a singular member in mammals, SLC4A11 (Cordat and Casey, 2008; Liu et al., 2015). In mice, due to different promoter regions *Slc4a2* can be transcribed as five different N-terminal variants: Slc4a2a, Slc4a2b_1_, Slc4a2b_2_, Slc4a2c_1_, and Slc4a2c_2_. Slc4a2a and Slc4a2b are ubiquitously expressed while Slc4a2c expression is restricted to the stomach. In OCs, *Slc4a2* expression is upregulated during differentiation in an NFATc1-dependent manner (Wu et al., 2008) and has been localized to the basolateral plasma membrane. *Slc4a2* knockout mice are viable and have been used as a model to study the role of the transporter in different tissues including the stomach, intestines, colon, biliary epithelial cells, testes, and ameloblasts (Rossmann et al., 2000; Medina et al., 2003; Lyaruu et al., 2008; Gawenis et al., 2010; Concepcion et al., 2013).

In bone, four independent studies have investigated the impact of *Slc4a2* deletion on skeletal homeostasis in mice. The first by Wu et al. (2008) assessed the bone phenotypes of mice globally lacking all *Slc4a2* isoforms. In this study *Slc4a2*-deficient mice were shown to manifest osteopetrosis with growth retardation and clubbing of the long bones. The authors went on to show that this disruption in bone homeostasis was related to a cell-autonomous defect in OCs and concluded that the primary function of Slc4a2 in OCs is to facilitate bone resorption by suppressing OC apoptosis (Wu et al., 2008). The second, by Josephsen et al. employed a global deletion mouse model of *Slc4a2*. In this instance, the knockout mice presented with an osteopetrotic phenotype affecting the skull and long bones. This high bone mass phenotype was attributed to defects in OCs, which where morphologically enlarged and possessed rudimentary ruffled borders. Soon after, Jansen et al. (2009) confirmed the osteopetrotic phenotypes of long bones in *Slc4a2a-b* knockout mice. However, in contrast to global *Slc4a2*-deficient mice, calvarias of *Slc4a2a-b* knockout mice are indistinguishable from wild-type littermates implying partial compensation from untargeted Slc4 isoforms. More recent studies by Coury et al. (2013) used an OC-specific conditional deletion of *Slc4a2* in mice. In this study, specific loss of Slc4a2 in OCs corresponded with a high bone mass phenotype and dysfunctional OCs which exhibited impaired bone resorption capacity owing to a dysregulation of calpain-dependent podosomal disassembly (Coury et al., 2013). The importance of SLC4A2 is not limited to mice, but has also been shown to underpin the osteopetrotic phenotype observed in Red Angus cattle (Meyers et al., 2010). Taken together, these findings highlight the indispensable role for the SLC4A2 transporter in buffering HCO3^–^ / Cl^–^ exchange during OC bone resorption. A future challenge will be deciphering the exact contribution of each SLC4A2 variant in OCs as well as exploiting this transporter family for therapeutic targeting. A recent study demonstrating that the anion exchange inhibitor 4, 4′-diisothiocyano-2,2′-stilbenedisulfonic acid (DIDS) is capable of suppressing OC activity in a mouse model of wear-particle-induced osteolysis (Wu et al., 2018) lends ‘proof of principal’ toward this.

## SLC4A7

Solute carrier family 4 member 7 (SLC4A7), also known as NBC2, NBC3 and NBCn1, is responsible for the electroneutral cotransport of Na^+^ and HCO_3_^–^ with a stoichiometry of one for one (Pushkin et al., 1999; Choi et al., 2000). SLC4A7 is expressed in many human tissues including the kidneys and gastrointestinal tract where it localizes to basolateral and apical cell membranes and participates in net cellular acid extrusion. The transporter is predicted to homodimerise and is understood to have 14 transmembrane domains with a long intracellular N-terminal and short C-terminal presenting a PDZ domain. SLC4A7 possesses a dileucine motif in its cytoplasmic C-terminal tail indicating potential lysosomal residency supported by reports of co-localization with LAMP1. The C-terminal of the protein has also been shown to interact with RACK1. In addition, some putative binding candidates for this terminal have been identified and include LAMP1, LAMP2, AP2A1, AP2B1, Rab5A, 5B, 5C, 8A, 11, and SNX1, 2, 5, 6, 27 (Olesen et al., 2018). The PDZ domain of Slc4a7 has been shown to interact with NHERF-1, PSD-95, and CFTR. There is also evidence for interactions between the C-terminal PDZ domain and V-ATPase subunits in renal tissues (Pushkin et al., 2003) and CA2, consequently recruiting CA2 to the plasma membrane (Loiselle et al., 2004).

*Slc4a7* expression has been detected at both the mRNA and protein level in neonatal rat OCs and murine OC-like cells where it has been suggested to function as a key participant in M-CSF-induced cellular alkalinisation (Bouyer et al., 2007). Indeed, *Slc4a7* expression is upregulated during osteoclastogenesis and had been localized to the ruffled border membrane (Riihonen et al., 2010). Consistently, knockdown of Slc4a7 expression using a shRNA lentivirus-mediated delivery impairs OC bone resorption and cytosolic pH homeostasis but does not alter OC formation or survival. To date, *Slc4a7* knockout mice have been generated although their skeletal phenotype remains unknown (Lee et al., 2016).

## Calcium Transporters in Osteoclast Signaling, Differentiation and Function

Ca^2+^ is a universal second messenger required for cell signaling and plays multiple roles in OC formation, survival and activity (Robinson et al., 2010). It is liberated from bone during resorption by OCs, which, in turn, exposes the ruffled border membrane to high concentrations of the ion (up to 40mM Ca^2+^) (Silver et al., 1988). Aforementioned, some of this Ca^2+^ is removed by transcytotic mechanisms or through passive leakage from the resorptive compartment during OC migration. However, a portion of this solubilized Ca^2+^ is exchanged transcellularly across OC membranes via a number of structurally diverse calcium transport proteins embedded on the surface of the OC ruffled border and basolateral domain that are described here (Berger et al., 2001).

## Ryanodine Receptor 2

Being enriched in the OC plasma membrane, the Ryanodine receptor 2 (RYR2) was initially proposed to be the major Ca^2+^channel controlling Ca^2+^ influx in OCs, a position supported by several lines of biophysical, pharmacological and immunochemical evidence in the early 2000s (Zaidi et al., 1995, 1999; Baljit et al., 2002; Datta and Horrocks, 2003). The role of the RYR2 in OCs has been reviewed extensively in Robinson et al. (2010) and thus will not be discussed further herein. Instead, we will focus on the contribution of more recently identified transporters and channels that have been implicated in modulating the exchange of Ca^2+^ across OC membranes.

## ATP2B1 and ATP2B4

*ATP2B1* and *ATP2B4*, also known as *PMCA1* and *PMCA4* respectively, belong to the P-type Ca^2+^ ATPase family that maintains intracellular homeostasis by exporting Ca^2+^ from the cytoplasm to the extracellular space. These pumps are powered by the hydrolysis of ATP, with one Ca^2+^ ion removed from the cell for each molecule of ATP hydrolysed. Calmodulin increases the affinity of the proteins’ alternatively spliced Ca^2+^-binding site and also increases the rate at which the pump extrudes Ca^2+^ (Keeton et al., 1993; Tidow et al., 2012). *ATP2B1* and *ATP2B4* are highly expressed in OCs and transcriptionally regulated by NFATc1 (Kim et al., 2012). Both ATP2B1 and ATP2B4 pumps reside on the basolateral membrane of OCs where they have been proposed to function to extrude superfluous intracellular Ca^2+^ and thus prevent Ca^2+^-induced apoptosis. Studies by Kim et al. (2012) demonstrated that siRNA-mediated double knockdown of *ATP2B1* and *ATP2B4* led to a significantly reduced bone volume to trabecular volume ratio and a significant increase in OC surface to bone surface ratio using a mouse calvarial injection model. Genetic ablation of *Atp2b1* in mice is lethal during early embryonic development and thus far has precluded assessment of the skeleton (Okunade et al., 2004). Similarly, there has been no reported bone phenotype for *Atp2b4* knockout mice, however males are infertile owing to reduced sperm motility (Okunade et al., 2004; Schuh et al., 2004).

## TRPV4-6

In addition to Ca^2+^ ATPases, OCs possess an array of Ca^2+^ channels including several members belonging to the transient receptor potential (TRP) channel family. TRP channels are a class of 6-membrane-spanning helices ion channels primarily located on the plasma membrane of several mammalian cell types. They can be divided into Groups 1 and Group 2 channels; with each possessing their own subfamilies. TRP channels mediate several sensory functions including pain, temperature, taste, pressure, and vision but have also been linked to regulatory mechanisms. Trpv1-6 (Transient receptor potential cation channel subfamily V member 1 to 6) are members for the TRPV subfamily of Group 1 TRP channels. Several TRPV members including TRPV4, 5 and 6 are known to be expressed in OCs. *TRPV4* mRNA and protein expression has been reported to be upregulated during OC differentiation. *In vitro*, TRPV4 has been implicated in OC formation, with overexpression or knockdown of the channel correlating to increased and decreased OC numbers, respectively (Cao et al., 2019).

In comparison, TRPV5 is primarily expressed in the kidney, intestines and OCs where it plays a central role in the systemic regulation of Ca^2+^ homeostasis by facilitating: (i) Ca^2+^ reabsorption in the kidneys, (ii) absorption of dietary Ca^2+^ by intestinal cells, and (iii) the release of the ion by active OCs. The importance of TRPV5 in Ca^2+^ homeostasis is exemplified in *Trpv5* knockout mice, which exhibit reduced renal Ca^2+^ reabsorption leading to hypercalciuria-induced polyuria coupled with urine acidification (Hoenderop et al., 2003). The Ca^2+^ lost through urine in *Trpv5* knockout mice is compensated by hyperabsorption of dietary Ca^2+^, which, in turn, leads to reduced bone cortical and trabecular thickness. In these mice, serum peptide hormones (PTH), Ca^2+^ and K^+^ levels are normal despite increased 1,25-(OH)2D3, implying that the decreased cortical and trabecular bone thickness observed in *Trpv5* knockout mice is an indirect consequence of prolonged elevations in 1,25-(OH)2D3 (Hoenderop et al., 2003). However, studies by the same group later reported a cell-autonamous defect in *Trpv5*-deficient OCs. In OCs, Trpv5 localizes to the ruffled border suggestive of a role in modulating bone resorption. Closer inspection of the bones of *Trpv5* knockout mice revealed that loss of *Trpv5* was associated with increased OC numbers and an increase in the number of nuclei per OC. Moreover, *Trpv5* knockout OCs exhibited reduced bone resorptive capacity in line with a intrinsic cellular defect (van der Eerden et al., 2005). Further, alendronate has been shown to normalize the reduced bone thickness in *Trpv5* knockout mice (Nijenhuis et al., 2008). It is noteworthy that Econazole (Yan et al., 2011), TH-1177 (Landowski et al., 2011), and selected cannabinoids (Janssens et al., 2018) have been reported to modulate TRPV5 but have potencies in the mid-micromolar range and are not selective for TRPV5 over its close homolog TRPV6. Hughes et al. (2019) identified three novel TRPV5 inhibitors through structural-based virtual screening. One of which was selective for TRPV5 over TRPV6 as revealed through the identification of a previously uncharacterised TRPV5 binding site by cryoelectron microscopy (Hughes et al., 2019). The apparent importance of TRPV5 in OC function along with recent development of selective TRPV5 inhibitors makes this channel a potential candidate for the therapeutic modulation of bone homeostasis.

Finally, similar to *TRPV5*, *Trvp6* knockout mice manifest a low bone mass (osteopenia) phenotype owing to increased OC numbers, and hence a net increase in OC bone resorptive activity *in vivo* (Chen et al., 2014).

## Mucolipin-1

Mucolipin-1 (also known as TRPML1) is the first member of the transient receptor potential cation channel, mucolipin subfamily and is widely recognized as a major calcium release channel residing on lysosomal membranes (Vergarajauregui and Puertollano, 2006; Thompson et al., 2007). Mucolipin-1 plays critical roles in a variety of membrane trafficking processes such as retrograde trafficking of lysosomes to the trans-Golgi network, autophagic maturation and lysosomal homeostasis (Haoxing and Dejian, 2015; Scotto Rosato et al., 2019). This non-selective cation channel is modulated by changes in Ca^2+^ concentration, phosphoinositides and pH. Mutations in *MCOLN1* lead to Mucolipidosis type IV, an autosomal recessive neurodegenerative lysosomal storage disorder (Bargal et al., 2000). The importance of Mucolipin-1 extends to bone. Genetic deletion of Mucolipin-1 in mice correlates with impaired osteoclastogenesis, altered lysosomal homeostasis and attenuated OC bone resorption pointing to an indispensable and physiological role for this cation channel in bone remodeling (Erkhembaatar et al., 2017). For an extensive review see (Wang et al., 2014).

## ORAI1

ORAI calcium release-activated calcium modulator 1 (ORAI1), also known as CRACM1 and TMEM142A, is a plasma membrane localized Ca^2+^ channel. It associates with stromal interaction molecule 1 (STIM1), an endoplasmic reticulum transmembrane protein, to modulate Ca^2+^ cellular entry through a process termed calcium release-activated calcium (CRAC) (Lunz et al., 2019). Upon depletion of luminal endoplasmic reticulum Ca^2+^ stores, the CRAC assembly formed by ORAI1 and STIM1 opens to refill depleted stores. To achieve channel opening, the cytoplasmic portion of STIM1 must come into contact with the intracellular portion of ORAI1 after which ORAI1, in turn, allows extracellular Ca^2+^ influx into the cell to replenish stores. Studies by Zhou et al. (2011) reported a decline in both *ORAI1* and *STIM1* expression during osteoclastogenesis in human cells. Moreover, they found that siRNA-mediated knockdown of *ORAI1* decreased OC formation. The authors further demonstrated that pharmacological inhibition of the CRAC channel assembly prevented OC differentiation. Using a shRNA knockdown approach, Hwang and Putney (2012) similarly reported that depletion of *Orai1* impairs OC differentiation *in vitro* owing to a decreased in NFATc1 transcritpional activity and associated downstream genes. Importantly, *Orai1* knockout mice exhibit a clear skeletal phenotype (Robinson et al., 2012). *Orai1* knockout mice are anatomically smaller than their wild-type counterparts and show less post-natal growth. In addition, they exhibit tooth abnormalities and bone defects, including the existence of unresorbed cartilage remnants and decreased bone mineralization. Accordingly, OCs from *Orai1* knockout mice are morphologically smaller (mononuclear) and osteoblastic maturation was impaired. Thus, the skeletal abnormalities observed in *Orai1* knockout mice appear to arise from cell-autonomous defects in both OCs and osteoblasts highlighting a general role for this Ca^2+^ channel in bone homeostasis.

## NMDAR

The N-methyl-D-aspartate receptor (NMDAR) is a glutamate receptor and ion channel found on the plasma membrane of OCs whose physiological function has long been debated. The channel is composed of three subunits: NMDAR1 associates with one or more NMDAR2 and/or NMDAR3 subunits. The multiple receptor subunit combinations along with the existence of 8 different NMDAR1 splice variants allows diversity in glutamate receptor function (Spencer et al., 2007). When activated by the binding of glutamate and glycine, the NMDAR allows for the flow of Ca^2+^ across the membrane. At present the role of NMDAR in OCs physiology remains contentious with several conflicting reports in the literature (Spencer et al., 2007). For instance, Chenu et al. (1998) reported that MK-801, an NMDAR inhibitor, had no effect on adhesion or survival of OCs but caused the disruption of actin ring formation. In contrast, studies by Peet et al. (1999) found no effect of MK-801 on OC actin ring formation but instead showed that MK-801 prevented OC differentiation and bone resorption in a co-culture model *in vitro*. Further, Merle et al. (2003) reported that MK-801 inhibited osteoclastogenesis and that activation of NMDAR induced the nuclear translocation of NFκB. Thus, further study using *in vivo* models will be required to resolve the physiological contribution of this Ca^2+^ channel in OCs and bone homeostasis.

## P2RX7

ATP-activated Ca^2+^ channel Purinergic receptor P2X 7 (P2RX7), also known under the alias P2X7, is a member of the Purinergic receptors P2X group that mediate Ca^2+^ fluxes across membranes in response to extracellular ATP. Following activation, P2RX7 forms a homotrimeric pore that facilitates the movement of organic ions including N-methyl-D-glucamine, choline and fluorescent dyes such as ethidium and YO-PRO-12 across membranes (Alves et al., 2014; Sluyter, 2017). P2RX7 is the major PX2 family member expressed in OCs (Naemsch et al., 2001) and its role in skeletal and joint diseases has been extensively reviewed (Zeng et al., 2019). P2RX7 has been implicated in OC fusion in both mice (Ke et al., 2003) and humans (Pellegatti et al., 2011). In humans, the formation of OCs is inhibited by an anti-P2RX7 monoclonal antibody and by specific P2RX7 pharmacological antagonists A740003 and AZ11645363 (Pellegatti et al., 2011). There is also accumulated evidence implicating several P2RX7 SNPs in postmenopausal osteoporosis (Ohlendorff et al., 2007; Gartland et al., 2012; Jørgensen et al., 2012). In keeping with this position, genetic deletion of *P2RX7* in mice corresponds with a significant reduction in total and cortical bone content and periosteal circumference in femurs, and reduced periosteal bone formation and increased trabecular bone resorption in tibias (Ke et al., 2003). Consistently, Wang et al. (2018a) reported increased OC formation in *P2RX7* knockout mice compared to wild-type littermates following ovariectomy (OVX)-induced bone loss. Further, whereas both the cortical and trabecular bone volume fractions were significantly decreased in the tibias of *P2RX7* knockout mice compared to knockout sham controls at 6-weeks post-OVX, no statistically significant change was observed in the corresponding bone parameters in OVX and sham operated wild-type mice 6-weeks post-surgery (Wang et al., 2018a).

## TPCN2

Two pore segment channel 2 (TPCN2), also known as TPC2, is a member of the Two pore channel family of intracellular voltage-gated and ligand-gated cation-selective channels that enables Ca^2+^ ion release in response to NAADP binding. Intracellularly, TPCN2 localizes to lysosome-related organelles via a lysosomal targeting motif (Brailoiu et al., 2010; Schieder et al., 2010; Ambrosio et al., 2015) where it has been shown to contribute to the regulation of their biogenesis (Ambrosio et al., 2015). In OCs, *Tpcn2* mRNA expression is upregulated in response to RANKL and has been proposed to play a role in maintaining intracellular Mg^2+^ rather than Ca^2+^ homeostasis. At steady-state Mg^2+^ levels, siRNA-mediated knockdown of *Tpcn2* has been shown to impair OC differentiation and bone resorption *in vitro*. Conversely, under low Mg^2+^ culture conditions, OC formation was accelerated suggesting that Tpcn2 functions to regulates OC differentiation and activity in response to circulating Mg^2+^ levels (Notomi et al., 2012, 2017).

## SLC8A1-3

Solute carrier family 8 member A1, A2 and A3 (SLC8A1, SLC8A2, SLC8A3), previously known as NCX1, NCX2 and NCX3, respectively, are Na^+^/Ca^2+^ antiporters from the SLC8 family. All three SLC8A transporters are expressed in OCs and are reported to localize to the basolateral membrane. Of these transporters, SLC8A1 has received the most attention in OCs (Li et al., 2007; Albano et al., 2017). The use of chemical inhibitors or siRNA to inhibit SLC8A1 and SLC8A3 leads to reduced bone resorption *in vitro* (Moonga et al., 2001; Li et al., 2007). Using an OC-specific *Slc8a1* knockout mouse model Albano et al. (2017) reported that *Slc8a1* knockout OCs form normally but resorb significantly more bone than their wild-type counterparts *in vitro*. However, no obvious skeletal changes were detected in *Slc8a1* knockout mice at 3 months of age with only very minor differences appreciable at 6 months of age. The findings *in vivo* suggest that Slc8a1 plays an accessory but not essential role in OC Ca^2+^ homeostasis.

## Phosphate Handling in the Osteoclast

Inorganic phosphate (Pi) is an essential molecule for cellular homeostasis and is the major anionic component of hydroxyapatite. Therefore, in addition to Ca^2+^, OCs are exposed to high ambient concentrations of Pi during bone resorption. Although the exact Pi concentration liberated into the resorption pit remains to be established, it is predicted to reach upward of 20 mM (Kuno, 2018). High ambient Pi has been shown to influence OC differentiation and resorptive activity underlying the importance of appropriate Pi handling by the cell (Kanatani et al., 2003). It has also been hypothesized that part of the Pi released from bone may be utilized by the OC to maintain cellular ATP during the energy requiring cyclical processes of migration, attachment, and resorption (Gupta et al., 1997). Typically, Pi influx has been reported to require extensive V-ATPase activity and thus a large amount of energy. Evidence of the existence of Na^+^-dependent Pi transport systems in OCs was first demonstrated by Gupta et al. (1996) and later confirmed by the Miyamoto and Gupta laboratories who demonstrated the presence of Pi influx and efflux systems in OC-like cells derived from RAW264.7 macrophages and primary mouse OCs (Khadeer et al., 2003; Mikiko et al., 2005). The expression of several Na^+^-dependent Pi transporters have been assessed in OCs including SLC17A1 (previously NPT1), SLC34A1 (previously Npt2a or SLC17A2), SLC34A2 (previously Npt2b), SLC34A3 (previously Npt2c), SLC20A1 (previously Pit-1), SLC20A2 (previously Pit-2) and SLC37A3. Of these, SLC34A1, SLC20A1/2 and SLC37A3 have been functionally characterized in OCs.

## SLC34A1

Solute carrier family 34 member 1 (SLC34A1), previously known as NPT2a or SLC17A2 is an 80-90 kDa sodium cation/divalent phosphate cotransporter with a stoichiometry of three Na^+^ to one HPO_4_^2–^ (Forster et al., 1999). It resides on cell membranes where it preferentially cotransports Na^+^ and HPO_4_^2–^, although it has also been reported to transport Li^+^ as a driving cation (Olga et al., 2012; Schlingmann et al., 2016). SLC34A1 sequentially binds two Na^+^ cations, a HPO_4_^2–^ and a third Na^+^ before undergoing conformational changes allowing the release of the ions on the opposite side of the membrane (Forster, 2019).

Several independent studies have implicated a role for *SLC34A1* in OC differentiation, bone resorption and skeletal homeostasis albeit with conflicting reports (Gupta et al., 2001; Khadeer et al., 2003; Albano et al., 2015). On one hand, studies by Albano et al. (2015) reported that SLC34A1 is dispensable for OC differentiation and bone resorption. On the other, studies by Gupta et al. (2001) reported that *Slc34a1* knockout mice exhibit an age-dependent bone phenotype associated with a reduction in OC numbers that accompanied increases in bone formation. The precise reasons for these discrepancies is unclear but may reflect differences in the targeting approach and mouse strains used. Alternatively, they might point to extra-skeletal disturbances in Pi homeostasis influencing the magnitude of bone phenotypes observed. For instance, outside of bone, *SLC34A1* is highly expressed in the kidneys. Inactivating mutations in *SLC34A1* have been associated with idiopathic infantile hypercalcemia 2 (IIH2), a disease usually attributed to mutations in CYP24A1 (Schlingmann et al., 2011; De Paolis et al., 2019). Thus, a major role of SLC34A1 is to facilitate reabsorption of glomerular-filtered phosphate in the proximal tubule, with disruption of this transporter leading to hypophosphatemia. Mutations in *SLC34A1* result in surplus active vitamin D in the body. This vitamin D excess, in turn, increases calcium absorption into the bloodstream, resulting in hypercalcemia (Schlingmann et al., 2016). Consistently, global *Slc34a1* knockout mice manifest hypophosphatemia and hyperphosphaturia associated with hypercalcemia and hypercalciuria all of which are known to influence bone mass.

## SLC20A1 and SLC20A2

SLC20A1 (also known as PiT-1 and Glvr-1) and SLC20A2 (also known as PiT-2, Glvr-2 and Ram-1) are sodium-phosphate (Na^+^/Pi) electrogenic cotransporters that are widely expressed. These transporters share 60% of their amino acid sequence although their precise stoichiometry remains to be established (Kavanaugh and Kabat, 1996; Bai et al., 2000). In OCs, both SLC20A isoforms are constitutively expressed throughout osteoclastogenesis (Khadeer et al., 2003; Albano et al., 2015). Of these, SLC20A1 has been localized to the basolateral membrane of bone-resorbing OCs (Khadeer et al., 2003). Global genetic ablation of *Slc20a1* in mice leads to embryonic lethality. However, a *Slc20a1* variant mouse in which both alleles code for a gene with 85% reduced expression (knockdown) are viable but manifest multiple abnormalities that do not extend to bone (Beck et al., 2010; Bourgine et al., 2013). Interestingly, *Slc20a1* knockdown mice exhibit an upregulation of *Slc20a2* implying functional redundancy. *Slc20a2* knockdown mice, however, show no overt bone phenotype but rather manifest features consistent with familial idiopathic infantile ganglia calcification as observed in humans harboring mutations in *SLC20A2* (Wang et al., 2012; Jensen et al., 2013). Future generation of a double *Slc20a1-Slc20a2* OC-specific knockout model might prove valuable toward resolving the ambiguity around the physiological importance of these two Na^+^/Pi transporters in OC function.

## SLC37A3

Solute carrier family 37 member 3 (SLC37A3) is a little studied member of the SLC37 family of glucose-6-phosphate (G6P)/Pi antiporters (Chou et al., 2013; Cappello et al., 2018). SLC37A3 is highly expressed in neutrophils and the pancreas implying an important role for this transporter in these systems. SLC37A3 has been shown to localize to the endoplasmic reticulum but unlike other SLC37 family members does not transport G6P (Pan et al., 2011). Instead, SLC37A3 has been recently implicated in the translocation of nitrogen-containing bisphosphonates (N-BPs) from lysosomes to the cytosol (Drake et al., 2008). Using a CRISPRi-mediated genome-wide screen, Yu et al. (2018) identified SLC37A3 among the top genes to confer resistance to N-BPs. They further demonstrated that SLC37A3 interacts with ATRAID, a type I transmembrane protein on lysosomes, that stabilizes SLC37A3 expression thereby allowing the transport of N-BPs into the cytosol. In keeping with this position, deletion of *SLC37A3* and/or *ATRAID* conferred resistance to alendronate in OC-like cells derived from RAW 264.7 cells. While SLC37A3 appears to facilitate efflux of N-BPs from the lysosome, its endogenous substrate remains to be defined.

## Other Solute Carrier Transporters in Osteoclasts

Among the multitude of membrane transporters known to exist in OCs (Figure 2), a growing number of members of the solute carrier (SLC) transporter superfamily have recently been identified in OCs and implicated in bone health and disease. SLC proteins constitute a large group of transmembrane transporters spanning 65 gene families and having more than 400 putatively functional protein-coding genes (He et al., 2009; Schlessinger et al., 2013). These transporters are organized into families based on predicted protein sequence homology and predicted substrate specificity (Chou et al., 2013). Some of these transporters are ubiquitously expressed while others are a specialized feature of terminally differentiated cell types like neurons and OCs. All members of this superfamily have a preferred substrate and subcellular localization allowing for a vast number of varied exchanges across biological membranes intracellularly and with the extracellular environment (Pizzagalli et al., 2020). A number of diverse SLC transporters have been recently linked to OC function involved in the exchange of a range of fundamental biological substrates including amino acids (SLC7A5/LAT1, SLC9B, SLC17A7/VGLUT1), nucleosides (SLC29A3) and iron (SLC40A1 and SLC11A2) that are described briefly herein.

## Amino Acid and Nucleoside Transporters

Amino acids and nucleosides are utilized as the building blocks for proteins, DNA and RNA, respectively, as well as serving critical roles in a variety of cellular signaling functions including synaptic neurotransmitter in neurons (e.g., the glutamate transporter SLC17A7/VGLUT1). Dysregulation of amino acid and nucleoside transporters have been increasingly implicated in various human metabolic diseases as well as cancer pathogenesis (Kandasamy et al., 2018; Pastor-Anglada and érez-Torras, 2018). Thus, it is not surprising that the importance of these membrane transporters has been extended to OC formation and function.

## SLC7A5

The solute carrier family 7 member 5 (SLC7A5), also known as L-type amino acid transporter (LAT1), mediates the cellular uptake of phenylalanine, tyrosine, L-DOPA, leucine, histidine, methionine and tryptophan. SLC7A5 forms a heterodimer through the formation of a disulfide bond with the solute carrier family 3 member 2 (SLC3A2). The heterodimer functions as a Na^+^-independent large neutral L-amino acid exchanger where SLC7A5 is the transport component (Napolitano et al., 2015). Whereas the heterodimer localizes to the plasma membrane, it translocates to lysosomes upon heterotrimerisation with LAPTM4B. At the lysosomal membrane, the heterotrimer allows for entry of amino acids into the lysosomal lumen leading to the V-ATPase mediated activation of mTORC1 (Milkereit et al., 2015).

Slc7a5 has been linked to the pathogenesis of various cancers (e.g., highly proliferative breast cancer subtypes and small cell lung cancer) where it is thought to modulate cell proliferation (El Ansari et al., 2018). More recently, the role of Slc7a5 has been extended to bone homeostasis. Studies by Ozaki et al. (2019) demonstrated that *Slc7a5* is expressed in both OCs and osteoblasts, with expression of the transporter found to decline in preosteoclasts of ovariectomized mice. Conditional deletion of *Slc7a5* in OCs yields a low bone mass phenotype associated with an elevation in the bone resorption marker CTx, whereas bone formation markers remained unchanged. Moreover, loss of Slc7a5 accelerates osteoclastogenesis and bone resorption in an mTORC1-dependent manner that, in turn, contributes to the nuclear accumulation of the pro-osteoclastogenic transcription factor NFATc1.

## SLC9B2

Solute carrier family 9 member 2 (SLC9B2), also known as FLJ23984 or NHA2 and NHEDC2, is a 547 amino acid antiporter that regulates the exchange of extracellular H^+^ for cytosolic Na^+^ or Li^+^. SLC9B2 is highly expressed in OCs and is transcriptionally regulated by TFEB (Sardiello et al., 2009). This transporter possesses an N-terminal lysosomal targeting motif and accordingly is localized to late endosomes as well as the OC basolateral membrane (Pham et al., 2007; Battaglino et al., 2008; Hilton et al., 2008; Lee et al., 2008). Despite its abundance in OCs, *Slc9b2* knockout mice lack a prominent skeletal phenotype (Hofstetter et al., 2010). Further, OCs from *Slc9b2* deficient mice differentiate normally and are capable of bone resorption *in vitro*, suggesting that SLC9B2 is dispensable in OCs or may be compensated by a hitherto unidentified transporter that fulfills its function (Pizzagalli et al., 2020).

## SLC17A7

SLC17A7, also known as BNPI or VGLUT1, is an electrogenic Cl^–^ dependent glutamate transporter of the SLC17 family. SLC17A7 is best recognized for its function in mediating the uptake of glutamate into synaptic vesicles in neurons, with a preference for L-glutamate over D-glutamate (Voglmaier et al., 2006). The importance of glutamate in bone homeostasis has long been recognized (Serre et al., 1999; Hinoi et al., 2001; Skerry and Taylor, 2001; Mason, 2004). In OCs, glutamate is secreted through the process of transcytosis. Studies by Morimoto et al. (2006) were the first to demonstrate the existence of the glutamate transporter in OCs where it was localized to transcytotic vesicles. Using OCs from *Slc17a7* knockout mice they further demonstrated that whereas the number and morphology of *Slc17a7* knockout OCs are comparable to those derived from wild-type littermates, loss of the transporter corresponded with altered glutamate signaling and significantly increased OC bone resorption. Accordingly, *Slc17a7* knockout mice exhibit reduced bone mass at 4 months of age (Morimoto et al., 2006).

## SLC29A3

Solute carrier family 29 member 3 (SLC29A3), also known as ENT3 or FLJ11160, is one of the four members of the SLC29 family. It is highly expressed in macrophages and OCs and encodes an equilibrative nucleoside transporter. SLC29A3 possesses an N-terminal dileucine motif necessary for its targeting to endosomal and lysosomal membranes (Baldwin et al., 2005). Various IDELS of *SLC29A3* have been linked to diseases including Faisalabad histiocytosis (FHC), H syndrome, sinus histiocytosis with massive lymphadenopathy (also known as familial Rosai-Dorfman disease), pigmented hypertrichosis and insulin-dependent diabetes have all been proposed to form part of a continuous ’SLC29A3 spectrum disorder’ (Morgan et al., 2010).

Campeau et al. (2012) identified SLC29A3 as the causative gene underpinning two cases of dysosteosclerosis, a rare bone sclerosing dysplasia. OCs derived from peripheral blood mononuclear cells isolated from these patients exhibited reduced differentiation and resorption capacity *in vitro* (Campeau et al., 2012). Howaldt et al. (2019) also reported sclerosing bone dysplasias in two patients with SLC29A3 mutations.

## Iron Transporters

In addition to Ca^2+^ and Pi, iron (ferritin) is an important element for OC differentiation and bone homeostasis. This is exemplified in patients with iron overload conditions such as hemochromatosis, thalassemia and sickle cell disease, which frequently suffer osteoporosis and associated fragility fractures (Guggenbuhl et al., 2005; Fung et al., 2008). Hemochromatosis, in particular, has been reported to arise from mutations in *SLC40A1* (Zhang et al., 2017; Ka et al., 2018; Yin et al., 2019). SLC40A1, also known as Ferroportin 1 and SLC11A3, is a 571-amino acid transporter localized to cell membranes where it functions to facilitate iron export (Hentze et al., 2004).

## SLC40A1

Global *Slc40a1* knockout, myeloid lineage-specific (*LysM*-Cre) and mature OC-specific (*Ctsk*-Cre) conditional *Slc40a1* knockout mice have been generated and characterized by several independent groups (Wang et al., 2018b; Pereira et al., 2020). Global deletion of *Slc40a1* in mice leads to embryonic lethality (Donovan et al., 2005). On the other hand, myeloid lineage-specific and mature OC conditional *Slc40a1* knockout mice are viable but exhibit mild or little bone phenotypes, respectively, which also depend on the sex, age and Cre-driver employed. For instance, *LysM*-Cre *Slc40a1* conditional knockout mice exhibit decreased trabecular bone mass of both long bones and vertebrae in female, but not in male littermates at 2-months of age. By comparison, specific deletion of *Slc40a1* in the late stages of OC differentiation by *Ctsk*-Cre has no obvious effects on either trabecular or cortical bone mass of both the long bones and vertebrae, suggesting that the importance of *Slc40a1* in OC bone remodeling occurs indirectly through myeliod projenitors. In keeping with this position, *Slc40a1-*deficient OC precurors (from female mice) have increased intracellular iron accumulation and exhibit accelerated OC formation *in vitro* but this was not observed in *Ctsk*-Cre conditional *Slc40a1* knockout mice.

In contrast, independent studies by Pereira et al. (2020) recently reported a high bone mass phenotype in 16 week-old female and male *LysM*-Cre *Slc40a1* conditional knockout mice associated with reduced OC formation *in vivo*. While the precise reasons for these discrepancies warrant further study, it may reflect differences in the age at which mice were phenotyped and the strain of Slc40a1 floxed mice investigated (C57BL/6N-Slc40a1tm1c(EUCOMM)Hmgu/H in the Pereira study compared to 129S-Slc40a1tm2Nca/J by Wang et al., 2018b) (Pereira et al., 2020). Regardless, together these findings highlight a role for Slc40a1 in the regulation of bone structure and strength via direct actions in the macrophage-OC lineage. In keeping with the importance of Slc40a1 in iron handling during OC differentiation, hepcidin, an upstream modulator of Slc40a1 has also been reported to modulate osteoclastogenesis *in vitro* (Xie et al., 2016).

## SLC11A2

Finally, like SLC40A1, the SLC11A2 transporter (also known as DMT1) has been shown to regulate iron uptake (Knutson, 2017). Currently, very little is known about the role of SLC11A2 in OCs. Nonetheless, at steady-state conditions, the expression of *SLC11A2* has been shown to be upregulated during OC maturation. Conversly, the expression of *SLC11A2* is downregulated in mature OCs following addition of exogenous iron to cell culture media *in vitro* (Xie et al., 2016).

## Therapeutic Potential of Membrane Transport Proteins for Metabolic Bone Diseases

Over the past few decades, the advent of genomic sequencing coupled with genome-wide association studies has rapidly expanded our knowledge of the number of membrane transport proteins underpinning the pathophysiology of various human diseases ranging from cancer to metabolic diseases such as obesity, type 2 diabetes and osteoporosis (reviewed extensively in Lin et al. (2015a), Zhang Y. et al. (2018), Schumann et al. (2020)). Not surprisingly, unraveling the substrates transported and molecular mechanisms regulating transporter activity has been the focus of intense scientific and pharmaceutical inquiry in recent years, in pursuit of lucrative therapeutic targets against these critical yet largely untapped protein superfamilies (ésar-Razquin et al., 2015). In fact, outside of G-protein coupled receptors (GPCRs), the most intensively studied drug targets, membrane transport proteins constitute the largest but most understudied group of potential new drug targets (Schumann et al., 2020). For example, more than 30 transporters of the SLC superfamily have recently been presented as current, prospective, or potential drug targets (Lin et al., 2015b; Schumann et al., 2020; Wang et al., 2020) and spawned strategic partnerships between pharmaceutical industry and academia, the largest conglomerate called RESOLUTE (Research Empowerment on Solute Carriers) (Pizzagalli et al., 2020). In addition, multiple drug classes targeting membrane transporters have already reached the market, with several additional drugs in phase II clinical trials, many aimed at targeting metabolic diseases (Lin et al., 2015b; Zhang Y. et al., 2018; Schumann et al., 2020).

Osteoporosis, defined as low bone mass associated with skeletal fragility and increased bone fracture risk (Lane, 2006), is by far the most prominent of all OC-mediated metabolic bone diseases. Frontline treatments for osteoporosis use remedies aimed at reducing further bone loss termed ‘anti-resorptive’ agents such as bisphosphonates and antibodies directed against the key OC differentiation cytokine RANKL (i.e., Denosumab) (Langdahl, 2020). Alternate treatments that help stimulate new bone formation (anabolic agents) are also available but the approved arsenal is small. These include parathyroid/parathyroid-related peptide hormones (PTH/rP) and the recently approved anti-sclerostin therapy “EVENITY” (Romosozumab-aqqg). While each drug is capable of building new bone, they remain (i) expensive; (ii) require subcutaneous injection (PTH/rP-daily; EVENITY-monthly); (iii) have limited efficacy (PTH/rP ∼18-months; EVENITY 12-doses, switching to standard anti-resorptives thereafter); (iv) carry increased treatment risks (PTH/rP-osteosarcoma; EVENITY-cardiovascular) and thus; (v) are restricted to patients with the most severe osteoporosis. Similarly, despite several anti-resorptive drugs on the market, issues remain concerning patient compliance and rare but unwanted side effects such as osteonecrosis of the jaw or atypical sub-trochanteric femoral fractures with prolonged use (Langdahl, 2020). Moreover, current anti-resorptive agents suppress OC differentiation and survival. This results in arrested bone turnover, as OC help regulate bone remodeling by communicating with osteoblasts and encouraging their recruitment, a process termed ‘coupling’ (Sims and Martin, 2020). Therefore, there remains an unmet need for alternative and better next-generation therapies for which membrane transporter proteins may serve as a promising and tractable drug target.

As detailed above, OCs possess a multitude of membrane transporters, many that are indispensable for OC formation and function. However, despite their obvious importance, to date very few have been targeted therapeutically for OC-mediated diseases. This is partly due to the technical limitations when designing inhibitors against integral membrane proteins. First, membrane transporters are very difficult to express and purify biochemically and thus the majority lack available high-resolution crystal structures required to inform the design of small molecule inhibitors and channel blockers. Second, there remain many membrane transport proteins for which the substrate transported is unknown, i.e., ‘orphans’ making drug targeting difficult. Third, the development and delivery of specific inhibitors that can readily penetrate OC membranes and target to specific intracellular transporters also remains a considerable challenge. This is perhaps best exemplified by drugs targeting the V-ATPase complex. Despite having attracted intense interest by bone researchers and spawned the development of a vast number of pharmacological V-ATPase inhibitors (summarized in Table 2), so far all have lacked targeting specificity to OCs and bone, precluding there clinical use for the treatment of OC-mediated diseases like osteoporosis. Similarly, given the high expression levels of CLCN7 in OCs and its indispensable role in bone homeostasis, CLCN7 has also received considerable attention as a potential therapeutic target for the treatment of osteoporosis. However, thus far pharmacological compounds targeting CLCN7 have been limited to preclinical studies owing to safety concerns regarding their extra-skeletal effects on retinal cells and the central nervous system given that CLCN7 knockout mice also manifest severe retinal and neuronal degeneration (Li et al., 1999). Nonetheless, CLCN7 inhibitors have been trialed successfully *in vitro* and in ovariectomized rat models and found to attenuate bone resorption while maintaining bone formation rates (Chalhoub et al., 2003; Valenzuela et al., 2013).

**TABLE 2:** V-ATPase inhibitors, targeting selectively and their impact on osteoclasts *in vitro* and *in vivo.*

V-ATPase inhibitor	Target	Selectivity	*In vitro*	*In vivo*
Bafilomycin	V_0_c V_0_a	Low	Widely used in experimental cell biology. Prevents OC resorption, and inhibits endocytosis and apoptosis (Xu et al., 2003)	Prevents OC resorption. Toxic upon systemic administration due to low OC specificity
Concanamycin	V_0_c	Low	Inhibits OC resorption and contributes to apoptosis (Okahashi et al., 1997)	Toxic due to low OC specificity
SB242784	V_0_c	High	Inhibits human OC resorption (Nadler et al., 1998)	Limits bone loss in thyroparathyroidectomy and ovariectomy rats (Visentin et al., 2000)
Iejimalides	V_0_	Low	Iejimalides A and B inhibit OC formation and cellular acidification. They also have anti-tumor activity (Kazami et al., 2006; McHenry et al., 2010)	
FR167356	Unknown	V-ATPase-High OC-Low	Seven-fold greater inhibitory effect of cell membrane V-ATPase than lysosomal V-ATPase. Inhibits resorption when accompanied by PTH, IL-1, and IL-6 (Niikura et al., 2004, 2005b)	Dose-dependently reduces retinoic acid-induced hypercalcemia in thyroparathyroidectomy and ovariectomy rats (Niikura et al., 2005b)
FR202126	Unknown	High	Inhibits resorption in the presence of IL-1, IL-6 and PTH (Niikura et al., 2005a)	Inhibits resorption (Niikura et al., 2005a)
FR177995	Unknown	Low	Inhibits resorption and does not affect OC numbers (Niikura et al., 2007)	Administration dose-dependently improves the BMD of the distal femur in adjuvant-induced arthritic rats (Niikura et al., 2007)
Diphyllin	Unknown	Low	Inhibits lysosomal acidification (Sorensen et al., 2007)	
KM91104	V_0_a3-V_1_B2 interaction	Medium	Inhibits OC formation and resorption (Kartner et al., 2010)	
Enoxacin Bis-enoxacin	V_1_B2-actin interaction	Low	Enoxacin dose-dependently inhibits OC formation (Toro et al., 2012). Bis-enoxacin also inhibits osteoclastogenesis and resorption	Bis-enoxacin inhibits resorption (Toro et al., 2013; Zhang Y. et al., 2018)
Salicylihalamide A Saliphenylhalamide	V_0_ (Xie et al., 2004)	Low	Saliphenylhalamid inhibits OC differentiation	Saliphenylhalamide inhibits OC resorption in a titanium particle-induced osteolysis mouse model (Qin et al., 2012a)
Artemisia capillaris extract	Unknown	Low	Inhibits differentiation and resorption by mature OCs	
Luteolin	V_0_a3-V_0_d2 interaction	Low	Controversial (Lee et al., 2009; Crasto et al., 2013)	Has a protective effect against ovariectomy-induced bone loss (Kim et al., 2011)

We anticipate therefore that new and enabling technologies, together with increased recognition of membrane transport protein importance, will bring about significant advances in the ability to develop new and selective next-generation therapies targeting specific OC membrane transport proteins that may serve as an alternative to conventional anti-osteoporosis therapies.

## Summary and Perspectives

In this review, we have summarized the ‘ins and outs’ of the main families of membrane transport proteins that regulate OC homeostasis. Briefly, the well-established V-ATPase proton pump is responsible for acidification and the formation of a membrane potential at the ruffled border that is neutralized by Cl^–^ transport while CA2 and SLC4 family members offer an internal buffering system for H^+^ and Cl^–^ levels. Mass release of Ca^2+^ by the bone is handled by a disproportionately large number of Ca^2+^ channels compared to only a few known Pi handling proteins. To date, intensive research has focused on understanding the transport of H^+^, Cl^–^ and Ca^2+^ across OC membranes, whereas our understanding of other small molecule transporters, including secondary active transporters (e.g., SLCs), remains comparatively underrepresented.

In spite of considerable progress over the past few decades toward the identification and expansion of the OC membrane transport protein inventory, very few transporters have been characterized functionally in OCs. This is, in part, due to several inherent challenges encountered when working with proteins embedded deeply in cellular membranes, which are not readily accessible to standard biochemical methods, together with a giant cell that is notoriously impervious to conventional cell biology manipulations such as transfection. Further, attempts to identify and characterize new membrane transport proteins in OCs have thus far been largely unsystematic, informed instead by research in more advanced polarized systems such as neurons and epithelial cells. It is likely, however, that OCs possess their own unique complement of membrane transport proteins necessary to fulfill their specialized requirement for rapid molecular exchanges during bone resorption. In this regard, an unbiased approach to identify OC-specific membrane transport proteins would therefore be a valuable future line of inquiry. Recent developments in ‘omics’ technologies coupled with genome-wide association studies may prove useful toward this goal.

## Author Contributions

AR contributed to the writing, tables, and accompanying figures. NP contributed to the conceptualization and writing and editing of the manuscript. PN contributed to the editing of the manuscript. All authors contributed to the article and approved the submitted version.

## Conflict of Interest

The authors declare that the research was conducted in the absence of any commercial or financial relationships that could be construed as a potential conflict of interest.

## Abbreviations

OC: osteoclast
V_1_: V-ATPase ATP-hydrolytic domain
V_0_: V-ATPase proton-translocation domain
BMD: bone mineral density
ARO: autosomal recessive osteopetrosis.
